# Pathways to greater government accountability for breaches of their obligations in relation to doping in sport: A legal analysis

**DOI:** 10.3389/fspor.2023.1129390

**Published:** 2023-02-27

**Authors:** David Pavot

**Affiliations:** Professor of Law, Research Chair on Anti-Doping in Sport, Department of Marketing, Business School, University of Sherbrooke, Sherbrooke, QC, Canada

**Keywords:** doping, international law, responsibiity, states, governement, UNESCO, WADA, international court of justice (ICJ)

## Abstract

The State doping scandal in Russia has highlighted a major discrepancy in the fight against doping in sport: on the one hand, the signatories of the Word Anti-Doping Code (federations, NADO's, etc.) are subject to a very strict regime and incur serious sanctions, while on the other hand, States, when they massively violate the rules, do not risk very important consequences in international law. For example, high ranking officials as well as the Russian state apparatus have not been affected with a few exceptions such as the Moscow antidoping laboratory. The aim of this opinion paper is to present a reflection on the different avenues that could be envisaged to make governments more accountable, especially as work is underway on the topic. The development of a true government accountability regime would allow the system to be more balanced.

## Introduction

For more than 20 years now, governments and the sport movement have been working together to build a robust and coherent global antidoping system. The development of the fight against doping was first initiated by the world of sport with the creation of WADA and the World Anti-Doping Code. The community of States joined this movement with the adoption, under the aegis of UNESCO, and then the entry into force in 2005 of the International Convention against Doping in Sport. This Convention is now the most widely ratified (191 States) of those administered by UNESCO (1). In international law, this is a rather innovative institutional construction. On the one hand, a foundation under Swiss law, WADA, which can be qualified as an international non-governmental organization (2), was created in 1999 and has been delegated strong regulatory powers with respect to the Olympic movement and athletes. On the other hand, an international convention, under the aegis of UNESCO but providing for a very flexible framework binding on States, subsequently completed the system in 2005.

Following the Sotchi 2014 Games, the declarations of the Stepanov couple and the former director of the Moscow laboratory, Grigory Rodchenkov, led to the establishment of an international commission of inquiry chaired by Richard McLaren, which found the existence of a system of state doping, notably involving the concealment of positive tests (3). A series of consequences followed: suspension of the Moscow anti-doping laboratory by WADA, prohibition for certain athletes to participate in international competitions, participation in the Olympic Games under a neutral banner, etc. However, although the laboratory and the Russian anti-doping agency (RUSADA) can be seen as agents of the Russian State, the Russian government has never incurred sanctions.

The Russian case is a perfect illustration of the regulatory asymmetry of the international antidoping regime in sport. Indeed, while the signatories of the Code as well as the athletes are subject to a very strict regime, the governments, bound by the Convention, are not the object of a dedicated responsibility system. In this sense, at the last Conference of Parties to the International Convention against Doping in Sport, President Banka noted that “governments are responsible for meeting the requirements of the Convention and must be held accountable for the commitments they have made in this regard. However, today there is no robust process under the Convention where governments face consequences for non-compliance” (4).

It therefore seems important to fill this gap to restore confidence in the system and not allow governments’ behavior to go unpunished in the event of massive violations of the rules. Thus, work has begun to address this issue: for example, at the WADA 2022 symposium (5), a panel of experts discussed the question, while UNESCO has just set up a working group dedicated to government accountability.

The purpose of this article is to present the different routes offered by international law while examining the contours and nature of responsibility in international law.

## International state responsibility and doping in sport

### The search for an obligation on states

The international responsibility of States was codified in 2001 in the International Law Commission's (ILC) articles on internationally wrongful acts (6). The basis of responsibility is the breach of an obligation by a State bound by it. Obviously, to be bound by a treaty obligation, the State must have ratified the treaty in question (6).

The framework text for clean sport is the International Convention against Doping in Sport, which came into force in 2005 and is administered by UNESCO which is the most ratified treaty (1). This treaty came after the creation of WADA in 1999 and the first Code. The objective was to ensure the participation of governments in clean sport. An analysis of the normative framework of the International Convention against Doping in Sport reveals that it contains only very light obligations: it is more of a text setting up goodwill soft law provisions aimed at collaboration, harmonization, funding, or education (7).

One of the few provisions developing an obligation for States is Article 3 of the Convention, which states that “[States undertake] to adopt appropriate measures at the national and international levels consistent with the principles set forth in the Code”. However, it appears from the discussions that the wording “principles of the Code” was chosen because of the opposition of certain States to simply mentioning “the Code” (8). However, the notion of principle is not deﬁned in the Convention and remains imprecise. However, this lack of definition can be seen as an advantage because of the scalability of the principles. On the other hand, while it can be readily agreed that a system developing a state doping system violates the principles of the Code, the limits would have to be tested: for example, could a State that does not sufficiently fight doping be targeted? Would a law such as the Rodchenkov Antidoping Act be covered by such a provision? An answer to such a question could make it possible to know the limits of the action of certain States, such as the United States, which have decided to renationalize (9) the fight against doping because of dissatisfaction (10) with the management of the Russian case, even if it means putting themselves on the bangs of legality (11, 12).

Once a breach of the obligation is established, it must be shown that the act is attributable to the State. This means that it must be shown that officials acted on behalf of the state and violated a state commitment Again, while in a case of State-sponsored doping this does not seem difficult, certain elements will need to be verified: for example, in many States the code is incorporated simply by action of the NADO. The imputation of such a violation remained uncertain.

### Is reparation for a violation of international law appropriate in the fight against doping in sport?

In international law, the preferred method of reparation is *restitutio in integrum* according to Factory of Chozow Case (13). In the case of doping in sport, it seems difficult to retain because it is illusory to think of correcting what has been caused by a state doping system. Therefore, another way could be satisfaction. The latter consists of the recognition, by an international tribunal, of the responsibility of the State. This means that the Court simply recognizes that the offending State has violated an obligation and that no reparation or pecuniary sanction is required. This form of reparation aims at conferring a moral benefit intended to compensate for the injury suffered by the violation of an international obligation, as the ICJ used in the Corfu Channel case in 1949 (14).

While in general international law, this method of reparation might appear sufficient, it might not be satisfactory to the antidoping community. Indeed, regarding the regime applied to the signatories of the World Anti-Doping Code, a parallelism with a sanction regime applicable to governments might be desirable. However, such measures are quite rare in international law. Indeed, international liability does not, in principle, have a punitive effect. One could, however, imagine measures such as the impossibility of playing the national anthem during competitions, of nationals of a State found guilty of violation to sit on certain committees, or of making this country ineligible to host certain competitions. This would require the articulation of several instruments, some of them new, and it does not appear that the thinking has reached that point.

### Identification and discussion of pathways

Hypothesis 1: an amendment to the International Convention against Doping in Sport

The natural route to greater state accountability would be through a revision of the core of their clean sport commitments, the International Convention against Doping in Sport.

The process of amendment, provided for in Article 33 (15), is not obvious and presents several difficulties. Firstly, it should be noted that a proposal for amendment may be submitted at any time to the Director-General of UNESCO by any of the States Parties to the Convention. The Director must then consult the other States Parties and if half of them support the amendment, he must present it to the COP: it would therefore be necessary today for 95 States, in addition to the submitter, to support the proposal. Once the amendment is presented, two third of the States (i.e., 128) would have to support it at the COP.

Once the amendment is adopted, States would still not be bound by it. They would have to ratify the amendment before it could be invoked against them. A State is under no obligation to ratify, nor is it required to ratify successfully. Furthermore, if an amendment was intended to develop a dispute settlement mechanism whether it is new or assigns jurisdiction to the International Court of Justice, it would be surprising if it provided for punitive sanctions for non-compliance. A priori, the only modality that could be included would be satisfaction that's to say limited to the recognition of the responsibility of the State sued. Indeed, with the number of States required to submit and adopt the amendment, only a watered-down version with a limited level of coercion could be accepted by the community of States in view of current trends in international law. Indeed, consensus or the meeting of a large number of governments is very difficult: we see this in other areas such as the WTO (16) or the environment (17, 18). However, the greatest risk of an amendment is that of having a two-tier convention: States refusing to ratify the amendment would remain under the current regime, while the others would be bound by the new mechanism. The system would become less clear. However, if a certain number of States ratified the amendment, the burden of proof would be reversed, and the recalcitrant States could feel some pressure.

Hypothesis 2: the implementation of an accountability system through the world anti-doping code

Increasing State responsibility could also be achieved through the World Anti-Doping Code. In the 2021 revision of the Code, Article 22 on government participation was changed from the imperative to the conditional. For example, article 22 (1) states that “Each government should take all actions and measures necessary to comply with the *UNESCO*”. This change was explained by the fact that States, non-signatories to the Code, could not be bound by commitments to which they had not consented.

At first glance, one might think that this change is regrettable, considering that any further revision should revert to the imperative. We do not share this analysis. Indeed, an overall reading of the States’ commitments and of Article 3 of the Convention shows that the States have undertaken to respect the principles of the Code. Therefore, any change in the verb tense in Article 22 is purely cosmetic.

The ability of governments to sanction through the Code should be clarified both institutionally and materially. Indeed, WADA should not have this capacity alone and should rely on an independent institution to determine whether a government has violated its obligations. Once this step of qualification of the facts is achieved, the Code could develop a diversified arsenal of sanctions. The simplest could be the impossibility for nationals of the responsible State to sit for a certain period on the various committees and bodies of WADA. One could also imagine that the Code provides for the impossibility for the country to bid for or host international competitions during a certain period. It would certainly be necessary to involve the International Olympic Committee and to develop a linkage with ongoing or already awarded competitions, but these avenues remain tangible.

Compared to the UNESCO Convention, such a regime could be put in place on a revision of the Code appears to be more flexible and could ensure the implementation of a uniform regime. The Code could not, however, establish a mechanism for the settlement of inter-state disputes or give jurisdiction to the ICJ. The measures that could be developed in the Code would be of a different nature but would be complementary or would make up for rigidities in international law. Obviously, these pathways are not mutually exclusive and could be implemented concomitantly, thus increasing the robustness of the system.

## The need for a court to characterize violations by states

Usually in international law, the responsibility of a State can be brought before a dispute settlement mechanism by a victim state. In the current state of international law, it would be possible to have recourse to the International Court of Justice, but the possibility of referring to it appears very limited because the way in which States recognize competence restricts the possibilities of access. Another solution would be to have recourse to an *ad hoc* arbitration mechanism, but the road to the creation of such an institution may be long and winding.

### Recourse to the existing: the International Court of Justice

Under its Statute, the International Court of Justice has contentious jurisdiction and advisory jurisdiction. The contentious jurisdiction is limited to States dispute and the advisory jurisdiction is only offered to UN institutions.

It would therefore be conceivable for a State to attempt to bring a case before the Court against another State in relation to a possible breach of its obligations in relation to doping in sport to obtain recognition of its responsibility. However, it is necessary for States to recognize the jurisdiction of the Court. Unfortunately, the International Convention against Doping in Sport does not contain a clause conferring jurisdiction on the Court or any other dispute resolution mechanism. In the absence of this, it is necessary to check whether the State has filed an optional declaration of compulsory jurisdiction under Article 36 para 2 of the Court's Statute (19) which does not exclude sports or doping disputes and allows the next State to bring a case against it. Finally, there is always the option of a post-dispute compromise, but this is increasingly rare in international relations. Applied to the recent case, neither Russia, which could have been prosecuted for its State doping system, nor the United States, whose Rodchenkov Anti-Doping Act compliance could have been verified, have a declaration issued under Article 36 para 2 (20) and refuse to sign compromises. Therefore, the use of this avenue seems to be uncertain.

Another option could be the consultative competence offered to UN bodies. In this sense, it would be quite possible for the Conference of Parties to the UNESCO International Convention to submit a request for an advisory opinion to the International Court of Justice. It does not seem to be excluded either that the Bureau of the COP could have such a competence, but this remains to be determined. The purpose of advisory opinions is to clarify a point of law and not to recognize the responsibility of a State. However, the Court is quite liberal in accepting questions put to it and it is not untrue that some of the questions put to it through advisory opinions in recent years could have been put for litigation purposes. Examples of this are the opinion on the Wall in Palestine (21) and the opinion on the Chagos Islands (22). In the latter, the Court, in the operative part of its opinion, ordered the United Kingdom to complete the decolonization process in the same way as it would have done for contentious purposes. The questions posed to the Court could range from whether a state doping system is lawful under international law to asking the Court to circumscribe the notion of World Anti-Doping Code principles.

Although the possibility of recourse to the International Court of Justice seems marginal, it is—to date—the only avenue available. There may be some criticism of the competence of the Hague judges in sports law or of the length of time it takes for a case to be decided by the Court. While the question of procedural delays is certainly not wrong, the question of the competence of the judges should be put into perspective, as they are generalists in international law who work daily in many fields. The establishment of international liability or the interpretation of certain treaty principles require such skills before having disciplinary skills in sports law.

### The creation of a new mechanism: towards a specialized arbitration tribunal?

At WADA's 2022 symposium, a proposal for the creation of an *ad hoc* arbitration mechanism to engage the responsibility of States in doping matters was developed. This idea aimed to present a voluntary mechanism. The suggested mechanism referred to an *ad hoc* arbitral tribunal, but its contours remained unclear. Would this tribunal have to decide on the responsibility of a government, leaving it to WADA and UNESCO to decide the consequences, or would it have an arsenal of sanctions at its disposal?

If this avenue seems interesting, several questions can immediately be raised. Firstly, such a body often needs a secretariat. Therefore, should this body be created under the auspices of UNESCO within the framework of the Convention with the rigidities of the amendment procedure? Another way would be to create an *ad hoc* arbitration mechanism by a treaty between the countries supporting the initiative. Immediately, the question of the secretariat would arise: should an existing institution offering such services, such as the Permanent Court of Arbitration, be used or should the mechanism have its own secretariat? Whichever option is chosen, cost would be a factor to be assessed. Secondly, how many arbitrators would there be? Would they be appointed by States in the event of a dispute, or should they be chosen from a predetermined list? The modalities of the composition of this list are again to be defined. In short, there are several issues that need to be resolved before such a mechanism can be considered. However, this is not impossible. Indeed, in the context of the WTO dispute settlement crisis, some States have agreed to develop a voluntary arbitration mechanism to circumvent the Appellate Body's blockage. Another problematic element is the nature of the obligations identified by this potential mechanism. Indeed, the creation of such a mechanism would not allow, prima facie, to circumscribe and specify the obligations of States. It would therefore necessarily, if the Convention does not evolve, have to interpret the existing rules and in particular the notion of principles of the World Anti-Doping Code.

## Conclusion

There seems to be a consensus today to implement the responsibility of governments when they violate their antidoping obligations in sport. The Russian doping scandal has highlighted the need to be able to mobilize State responsibility—which is not the case today—in an adequate manner. Indeed, the system cannot, on the one hand, be very strict regarding the signatories of the Code and the athletes, and on the other, lax regarding the States. However, choices will have to be made: should this be done through the Convention? Should the ICJ be used? Should an *ad hoc* dispute resolution mechanism be developed? Can the regime be supplemented by the Code? There are many ways to go and the choice will have to be made by the community of States. If things are to move forward and quickly, it would be urgent for a group of leading governments to carry this project forward so that present and future initiatives do not remain a dead letter. In political terms, a silo response developed by either UNESCO or WADA should be avoided. The responsibility of governments will only be achieved by States working together. Perhaps the next World Conference on Anti-Doping in Sport, scheduled for 2025, would be the ideal place to initiate a collective dialogue.
